# America’s opioid crisis: the need for an integrated public health approach

**DOI:** 10.1038/s41398-020-0847-1

**Published:** 2020-05-28

**Authors:** Carlos Blanco, Tisha R. A. Wiley, Jacqueline J. Lloyd, Marsha F. Lopez, Nora D. Volkow

**Affiliations:** National Institute on Drug Abuse, Bethesda, MD 20892 USA

**Keywords:** Diseases, Addiction

## Abstract

Continued increases in overdose deaths and recent declines in life expectancy call for need to adopt comprehensive public health approaches to the United States opioid crisis and to establish an infrastructure to avert future crises. Successfully addressing the challenges posed by the crisis requires a translational, integrated approach that combines the contribution of neuroscience, pharmacology, epidemiology, treatment services and prevention. It also is critical to integrate interventions across settings, including healthcare, justice, education and social service systems. This review highlights four interconnected themes: (1) social determinants of health and disease; (2) person-centered approaches for prevention and treatment; (3) bridging the gap between implementation science and practice; and (4) using data to build learning systems of care, relevant to public health approaches to address the opioid crisis. We discuss how across these four themes taking into account the influence of developmental factors on brain function and sensitivity to environmental stimuli including drugs, addressing the complex interactions between biological and social factors, and promoting an ongoing dialogue across disciplines and settings will help accelerate public health advances that are evidenced based and sustainable to address the current opioid crisis and avert future ones.

## Introduction

The steady increase in overdose deaths in the United States and recent declines in life expectancy has brought to the forefront the need to adopt comprehensive public health approaches and establish an infrastructure to avert future crises. This crisis, unabated to date, is fueled by increased abuse of synthetic opioids, a rise in deaths from psychostimulants, and low rates of treatment entry and retention. A public health approach, guided by advances in epidemiology, prevention, and treatment services, is essential to develop an understanding of the nature of the crisis and its evolution and to develop effective and sustainable responses. This public health approach should seek to understand who is affected; where they are affected; the trajectories, pathways, and consequences of opioid use and misuse and how these trajectories are changing; and the factors that mitigate these components. Also essential are strategies to prevent opioid misuse in particular and drug use in general, especially in at-risk populations, and to provide both effective and cost-effective treatment services to those with opioid use disorder (OUD).

Epidemiologic data have highlighted many facets that are important for prevention and treatment to address^1^. This includes understanding the complex relationships between prescription opioid use and the development of OUD, identifying risk and protective factors for OUD^2,3^, and understanding pathways from initial use of opioids to opioid misuse and OUD in different populations, including the role that misuse of legal and illicit substances has for priming subsequent misuse of opioids by themselves or in combination with other drugs (such as alcohol, psychostimulants, or benzodiazepines). However, there is a need to obtain more detailed data to identify subpopulations in need of targeted interventions and to develop and test more effective interventions. Although there are effective interventions to prevent substance use and misuse, these interventions are primarily targeted to younger age groups^4^ and, to date, they have shown only preliminary evidence for preventing opioid misuse^5^. At present, there are no known evidence-based interventions for preventing OUD in any age group. The etiology of OUD is multifactorial (as is the case for other substance use disorders), with individuals having different combinations of risk factors^2,6–8^. Interventions will need to address these heterogenous populations or, alternatively, different interventions will need to be developed for each population. Furthermore, there are populations at the intersection of prevention and treatment that may neither be identified nor provided treatment or intervention services. It is important to develop strategies for identifying and reaching these populations.

Successfully addressing these challenges requires an integrated approach that combines the contribution of knowledge derived from neuroscience, pharmacology, epidemiology, treatment services and prevention. It also is critical to integrate knowledge and resources across settings, including healthcare, justice, education and social service systems. This integration will require ongoing dialogue between research, policy and practice, i.e., a learning healthcare system^9^ or, perhaps more broadly, a learning system of care. OUD prevention and treatment must build on what we know about the epidemiology of substance use and substance use disorders (SUD) generally, and OUD specifically. Epidemiology must also build on insights from treatment and prevention research and practice. Achieving a learning system of care will be challenging, but necessary to successfully address the opioid crisis as it evolves and for addressing future drug crises. To date we have seen the evolution from prescription opioids, to heroin, then to synthetic opioids such as fentanyl and more recently psychostimulants with and without opioid combinations. In this review, we highlight four interconnected themes that cut across epidemiology, prevention and treatment services research relevant to public health approaches for addressing the opioid crisis and creating a foundation to mitigate future crises. These themes include (1) social determinants of health and disease; (2) person-centered approaches for prevention and treatment; (3) bridging the gap between implementation science and practice; and (4) using data to build learning systems of care. Across these four themes, we discuss the importance of addressing developmental factors, complexities, and dialogue across disciplines and settings for making meaningful advances (see Table 1).

**Table 1:** Overview of themes, current research findings, and opportunities for research.

Theme	Current research findings	Opportunities for research
Attend to social determinants and social context	Models of drug distribution differ across communities and can influence culture of drug use Nudges may influence prescriber behavior Peer intervention models^20^ and role of peers in recovery	Establish novel ways of collecting data to better understand how drug distribution influences drug use and can provide opportunities for prevention and treatment Apply modeling to data on social contextual risk and protective factors to predict OUD, to improve prevention and treatment strategies, and guide policy Explore how social networks can be used to identify, access and engage high-risk, difficult to reach populations for prevention and treatment
Emphasize a person-centered approach	Exposures across different stages of development confer unique risk and motivation for engagement and continuation in opioid misuse^41,43^ Patient perceptions of participation in treatment decisions regarding MOUD can enhance outcomes^103^	Uncover the most salient targets for intervention at different stages of development and trajectory of use Use optimization adaptive intervention designs to tailor strategies for prevention and treatment interventions to meet the needs of diverse at-risk groups
Bridging the gap between implementation science and implementation practice	Implementation science is identifying effective implementation strategies for evidence-based practices. Even effective interventions may not be sustained if not designed to fit well into existing clinical practices^33^	Consider scalability and the role of intermediary organizations in Evidence-Based Practice (EBP) scaling Take a user-centered design perspective that considers patients, providers, and scalability
Use data to build cross-system collaborations and learning systems of care	Policy focus (e.g., reduce number of prescriptions, target people with OUD) can have significant impact on the broader use and outcome profile ^86^ Evidence-based community coalition-based implementation systems, provide frameworks for utilizing community level data to inform selection, implementation, and evaluation of prevention programs (e.g., CTC, PROSPER)	Use modeling of responses to develop a portfolio of interventions with the greatest positive public health impact Utilize evidence based-implementation frameworks to engage community and system stakeholders and in the planning, implementation and evaluation of comprehensive prevention and treatment programming in a community

## Theme 1: Attend to social determinants and contexts of opioid use

Because of the urgency of the crisis, most efforts to date have focused on treatment strategies targeted to individuals. By contrast, the social context and social determinants of health have received much less attention, in part because efforts to address them may not yield immediate results but instead benefits will be observed in the long term. Several lay public publications have identified the breakdown of the social fabric of small rural towns across America^10^, the loss of meaningful social connections^11^ and the concept of diminished hope and purpose as important influences in the development of the opioid crisis. The social determinants of the epidemic have led to its characterization as a “disease of despair”^12^ along with suicide and alcohol-induced liver disease, all of which have increased in the past two decades. However, questions pertaining to how loss of hope and purpose and other social determinants have contributed to the opioid crisis and how they continue to influence its evolution have yet to be adequately addressed.

Within this theme, we highlight four areas of opportunity:

1. Develop a deeper understanding of the social contextual circumstances of substance use and misuse at the individual and population levels.
2. Understand the role of social context in relation to engagement in prevention and treatment services and the role of social supports.
3. Take a developmental perspective in attending to the role of family, peers, and social identity.
4. Explore the role of technology as a setting for social interactions and interventions.

### Understanding the social context of opioid misuse

Much of the data on the consequences of opioid use come from national population studies^7,13^. These data are often used to guide decisions about the structure and delivery of prevention and treatment services. This method of gathering information may not adequately recognize the nuances of the current opioid crisis because of declining participation rates in these types of studies and lack of accessibility to marginalized populations, such as drug users. These studies often exclude the more detailed questions that capture data necessary to identify targets for intervention. Novel and comprehensive ways of collecting population-level data are needed to focus resources in the most effective direction. Information captured in large-scale epidemiological datasets should be supplemented by in-depth qualitative studies to capture the complex nature of opioid misuse and the evolution from prescription opioid misuse to injection of heroin, synthetic opioids or drug mixtures. This approach can facilitate a better understanding of the social context of substance use.

Ethnographic research on how the social context of drug markets influence drug use behavior creates opportunities for targeted intervention. For example, Cicero and colleagues showed that models of drug distribution (i.e., “retail models”) differ across communities. In some communities, such as Philadelphia, drugs are often distributed via outdoor local dealer markets, whereby drug dealers offer free samples of powder heroin and focus on developing unique brands. In contrast, San Francisco black tar heroin is distributed through private drug sale interactions across the city, without branding or free samples. In this environment, dealers can be harder to access, which leads users to pool resources to share a larger purchase, facilitating use in groups. In both cases users see overdoses as an “advertisement” for quality, and accessing the same batch is facilitated by an open market such as the one in Philadelphia. Overdoses are viewed by some drug users as indicators of high quality and purposedly sought out, highlighting how understanding the motivations of people who use drugs is needed to prevent overdoses. For such indiivudals harm reduction strategies may represent an essential approach and an eventual gateway to treatment.

### Addressing social context in settings of care engagement

Prevention interventions can be delivered in diverse settings, including schools, community, healthcare, justice, and other social service settings^4^. Treatment for OUD has traditionally been delivered in the specialty behavioral health treatment system, which largely exists outside the rest of the medical system. Methadone is highly regulated and almost universally delivered in the specialty behavioral treatment system, where as buprenorphine and naltrexone, can be delivered in general medical settings. Increasingly, healthcare settings such as primary care and emergency departments are seen as a key touchpoint for both prevention and treatment services. In parallel, there is increasing recognition that justice settings offer a unique opportunity for providing OUD treatment. Promising approaches to treatment in justice settings include continuing or initiating of medications for opioid sue disorder (MOUDs) while people are incarcerated^14,15^, or adapting drug court models to incorporate MOUDs. It is important to consider the role of social context across all of these settings for pregnant women, ethnic minorities, and sexual and gender minorities all have specific needs that relate to their social identity and context.

As highlighted in the CDC opioid prescription guidelines in 2016^16,17^ and the National Pain Strategy^18^, addressing prescribing behavior has great potential for preventing prescription opioid misuse. Indeed, significant progress has been made in recent years in addressing prescribing of prescription opioids (e.g., widespread implementation of prescription drug monitoring programs [PDMPs]). A promising approach targeting the social context of care settings is use of social cues as part of behavioral “nudges”^19^ to improve opioid prescription practices.

Most interventions primarily target individuals, with little emphasis on the broader social context that surrounds them. Interest in treatment may be influenced by the experiences of peers^20^ and peers can potentially be utilized to enhance the effectiveness of existing interventions. In response to opioid and HIV/AIDS crises, models that include peer and social network intervention components have emerged and been scaled quickly^21^. A significant body of evidence supports the influence of social network-based strategies and interventions on HIV prevention and treatment outcomes, specifically for people who use drugs, including those who inject them^22,23^. Though these models are intuitively appealing, peer intervention models for addressing opioid misuse and OUD are largely untested and often implemented in an ad hoc manner. Unless these models are rigorously studied, it will be difficult to identify and replicate successful strategies. Such studies will inevitably investigate whether patients benefit from this kind of intervention; however, they should examine possible iatrogenic effects on peer interventionists^24^. Such models should pay attention to the needs of peer interventionists, with emphasis on addressing vulnerability to relapse and secondary trauma, as well as training needs and compensation.

### A developmental perspective of social context

Social context must be considered from a lifespan developmental perspective. The roles of families, schools, romantic partners, and social peers evolve during periods of developmental transition, and may differ across subpopulations. Vulnerabilities may emerge as individuals transition across life stages and between care systems (e.g., from receiving care in pediatric settings to adult settings).

Many studies of adolescents have focused on family-based approaches to prevention and treatment and shown that family-based interventions reduce both initiation and existing substance use in adolescents^4,25,26^. The evidence base is well established for general drug prevention interventions, primarily for youth, delivered to families, in schools, and community settings^3^. Less established is the evidence for prevention interventions that specifically target opioid misuse and disorder. While opioid use is very low among teenagers and has been decreasing in the past decade, the transition into young adulthood reveals an increase in the incidence of opioid use and OUD that is among the highest we have ever seen. Thus the need for prevention strategies that focus on the transition into and in young adulthood.

There is an opportunity to capitalize on influences of social context as people transition into adulthood, both for prevention and treatment. Epidemiologic and ethnographic work is needed to expand our understanding of how social support systems accessible in emerging adulthood can be used towards prevention and treatment interventions and to support recovery. For example, positive social interactions and support networks can prevent people from progressing from substance use initiation to misuse and SUD, and can also facilitate recovery^27^. Social network strategies, targeted to social peers, are also powerful tools for preventing other harmful outcomes, such as HIV^22,23^. Another promising area for exploration is understanding how social identity is influenced by social context and evolves over the course of initiation, escalation to addiction and recovery^27,28^. These are all areas ripe for exploration where a transdisciplinary feedback loop could be beneficial in speeding discovery.

Vulnerabilities created during developmental transitions may lead to increased likelihood of substance use initiation, worsening of existing substance use, risk of psychiatric co-morbidities or a disruption in care or services. For example, vulnerabilities may be introduced during the transition to adulthood as individuals switch from a pediatric to an adult system of care. Young adults who are isolated and not connected with family, educational institutions, or other traditional service systems may be particularly vulnerable to falling through the cracks as they transition between systems of care. These young people may be homeless or in unstable housing, have limited education, lack health insurance and may not seek any type of health care or other services in traditional systems. Youth aging out of foster care and transitioning to independent living and those transitioning out of juvenile justice to the adult justice system are particularly vulnerable^29^. Similarly those with a mental disorder, particularly if untreated, are at significant risk for misuse of alcohol and other drugs^30,31^. There is a need for strategies for identification and linkage to appropriate services, to facilitate and support individuals during these transitions, and to ensure continuity of both prevention and treatment services, especially for individuals with mental disorders or with justice involvement^32^.

A developmental perspective is not solely focused on young adults, however. We advocate for a developmental perspective across the lifespan to understand how addiction develops and presents itself at the various stages of life, as discussed in the next section. Very little data exists, however, exploring the role of social context across developmental transitions in adulthood. Key developmental transitions in adulthood such as entering the workforce, becoming a parent, changing jobs, getting married, caring for aging parents, retiring, or loss of spouse are often marked by significant stressors and changes in the social context. The effect of such changes on substance use and addiction are largely unexplored in the context of both treatment and prevention.

### Technology as a setting for social interactions and intervention

Technology represents an important context to understand influences on substance use behaviors and as a platform for intervention. Studies analyzing social media posts have elucidated a complex interplay between motivations to use opioids and engage in treatment. Although technology is a major component of the modern social context, most studies continue to approach technology primarily as a facilitator or delivery platform of an individualized intervention. Far fewer studies have emphasized using interactions within social networks and on social media as part of a technological intervention. Studies that have included this, even as a minor component of a larger intervention package, have yielded findings that suggest that online platforms that facilitate peer support can be a feasible and effective intervention tool^33^. One recent study found that approximately half of patients engaged in substance use treatment expressed interest in receiving support and external monitoring of their social media account to prevent relapse^34^. Another study reported that more than half of patients continued to use a comprehensive mHealth intervention after 12 months^33^. Some individuals gravitate to technology-based peer support networks and online forums such as Reddit to seek help and information^35,36^. These technology-based venues provide opportunities for epidemiologic, prevention, and services research.

Moreover, these technological advances offer the opportunity to deliver prevention interventions at the population levels that would have heretofore been inaccessible. For example, inasmuch as lack of education increases the risk for SUD, one could conceive of interventions that used web-based resources to deliver education and training to expand job opportunities (i.e., https://opportunityamericaonline.org). No published study has evaluated the effectiveness of such an intervention for preventing SUD in high-risk populations or for facilitating treatment and recovery in those with SUD.

## Theme 2: Emphasize a person-centered approach

Person-centered care emphasizes patient values, needs and preferences in the delivery of care^37^, including prevention and treatment^38,39^. The overarching goal of a person-centered perspective is to provide the right intervention for the right person, at the right time, and to attend carefully to patient voices in the design or redesign of systems of care. To bring an end to the opioid crisis, we must understand what leads people to misuse opioids and other drugs, as well as understand protective factors of those individuals and communities less impacted^40^.

Within this theme we highlight four areas of opportunity:

1. Use a developmental framework to understand the evolution of opioid addiction.
2. Understand motivations for opioid misuse and service engagement.
3. Tailor prevention and treatment interventions to patient preferences.
4. Emphasize positive development and recovery.

### Developmental perspective on the evolution of opioid addiction

A developmental perspective is important for understanding the evolution of opioid use to misuse and OUD across the lifespan. For example, for young people, the most common source of opioids is opioid prescriptions from friends and family. However, young people may also initiate prescription opioid use to treat pain from acute injury, routine dental procedures, surgical procedures, or chronic diseases (e.g., sickle cell disease). For example, opioids often are introduced to student athletes after an injury^41^, increasing the risk for non-medical use of prescription opioids for a subset of these athletes. Some young people also initiate misuse of prescription medications for their rewarding effects and the belief that they are safer than illicit drugs^42^. These exposures in young people confer unique risks due to incomplete neurodevelopment.

Equally important is to understand the pathways on the other end of the developmental spectrum. Rates of non-medical use of prescription opioids have been increasing in adults ages 50 years and older, a population for which there have not been targeted public health efforts to understand the circumstances around engagement and continuation of opioid use^43^. Adults with a psychiatric disorder are more likely to be prescribed opioid medication for pain conditions^44^, which exposes them to the risk of OUD.

There are potentially important cohort effects in the opioid crisis. One study that examined generational differences among Millennials, Generation X, and Baby Boomers, found that successively younger generations were more likely than the older ones to use heroin after a period of non-medical use of prescription opioids. This finding did not differ by sex but was less pronounced among African Americans. In all generations, the use of both prescription opioids (non-medically) and cocaine was predictive of heroin initiation^45^. Another study demonstrated generational differences in exposure to drug cues and recovery information, as well as interest in using technology for recovery support^46^. Additional work is needed to understand how motivations for opioid misuse, continuation, discontinuation, and treatment engagement differ across generations, developmental periods, sex, and race/ethnicity.

### Motivations for opioid use and service engagement

Person-centered research also enhances our understanding of the interplay between individuals’ motivations for using drugs and motivations to engage in prevention interventions and treatment. For example, many people who use illicit opioids are exposed to fentanyl, though most do not report a desire for fentanyl^47,48^. A person-centered perspective may change how we interpret diversion of medications such as buprenorphine. Qualitative work suggests that much of buprenorphine diversion reflects attempts to self-treat OUD or stave off withdrawal by people who are unable to access buprenorphine through medical settings^20^. Other studies have demonstrated that patients may not feel that the treatment is adequately addressing their cravings, which could lead to a reinterpretation of outcomes such as low adherence or treatment dropout^49^. It is critical that we understand patients’ motivations for engaging (or not) in treatment^50,51^, including contribution of hopelessness and suicidality in risky opioid consumption^52^, as well as factors that deter their engagement including fear of discrimination or distrust of medications. This could lead to important insights about how to best utilize harm reduction approaches such as safe consumption spaces and fentanyl test strips^53,54^, and could lead to new approaches in treatment and prevention design and patient engagement and retention strategies.

### Tailoring prevention and treatment interventions

Variability in etiological pathways, severity, clinical presentation and patient preferences, suggests the need to develop personalized approaches to treatment and prevention. Precision medicine approaches suggest that in the future it may be possible to blend findings from neuroscience and genetics to prevent and treat OUD. To be truly person-centered, however, precision medicine should also address patient values, needs, and preferences, which are often overlooked in studies of prevention interventions and SUD treatment effectiveness. This perspective runs counter to a common approach in addiction treatment in which treatment is mandated for a patient’s “own good” with less regard for the patient’s perspective. Drug courts that mandate specific treatments with limited opportunity for input from the patient and involuntary commitment to treatment are prime examples of this approach, though it is important to note that some research has suggested that mandated treatment in some patients can increase treatment completion^55^. Nonetheless, a person-centered approach is consistent with a growing body of evidence about the value of shared decision making in medicine^56,57^.

There are three approved medications for OUD (Table 2), but little data to suggest which treatment will be most beneficial to a given patient. Often, decisions are made based on where a patient can get treatment—most facilities providing OUD treatment offer only one option^58,59^. When structured in this way, all patients receiving care within a given facility tend to receive the same intervention based on policy rather than on a dialogue between the patient and clinician about which medication is best suited to the patient needs. Although some best practices guidelines for treating OUD use the existing literature to draw conclusions about optimal approaches for tailoring OUD treatment^60^, there are limited numbers of relevant studies and considerable debate regarding how best to incorporate patient preferences in OUD treatment. Extant studies to date suggest that patterns of engagement in prevention services and treatment are complex. For example, some people with OUD find it difficult to initiate naltrexone, others are more easily engaged with it and yet others may be particularly vulnerable to relapse after a failed attempt at naltrexone treatment^61,62^.

**Table 2:** FDA-approved medications for OUD: availability and care settings.

Medication	FDA-approved formulation	Care setting
Methadone	Oral liquid	Federally-certified Opioid Treatment Programs (OTPs)
Buprenorphine	Oral (buccal/sublingual) Film*	Out-patient primary care Addiction Specialty Treatment Federally-certified Opioid Treatment Programs (OTPs)
	Pill*	
	Long-acting injectable*	
	Implant*	
Naltrexone	Oral	Out-patient primary care Addiction Specialty Treatment Federally-certified Opioid Treatment Programs (OTPs)
	Long-acting injectable	

^*^Requires additional training/certification.

A challenge is that designs that incorporate patient preferences can be difficult to execute, though there are some promising studies under way^63^. Sequential Multiple Assignment Randomized Trial (SMART) designs are one promising methodological tool for testing hypotheses about tailoring prevention and treatment interventions to patient needs and preferences^64,65^. In thinking about patient preferences, it is also important to bring a flexible perspective to intervention designs that leverage social supports^66^ including interventions to increase retention in medication for OUD (MOUD). On average most treatment programs have a 50% relapse rate at 6 months of initiation of MOUD and thus research to understand the intervention that might increase treatment retention and recovery for a given patient are needed.

We need to understand better how patients and their support networks seek information on where to find help^67^, as well as strategies for engagement in prevention interventions, including use of person-centered technologies^68^. Research lends surprisingly few insights into use of help-seeking behaviors to best connect patients to effective, high-quality services. For example, patients may also seek input from other patients, including through publicly available reviews of treatment providers. Though it is unclear whether patient-generated reviews are a good indicator of treatment quality, there is strong interest from patients and their families for this kind of information^69^. More research is needed on the relationship between patient perspectives on quality and other more traditional measures of quality.

### Emphasis on positive development and recovery

A person-centered perspective emphasizes an individual’s strengths in how we think about opioid misuse, addiction, and recovery. Much of the research on risk and resiliency for drug misuse and disorder has focused on risk and vulnerabilities rather than protective factors, resiliency, and building assets to promote positive development^70^. More research is needed on assets and resiliency, as well as strategies to promote positive development across time and in settings that are opportune for promoting healthy development.

This person-centered emphasis on strengths and resiliencies is also applicable to recovery. Existing long-term studies of SUD clearly demonstrate that relapse is a common experience among those achieving recovery^71–73^. And though abstinence has commonly been the metric for defining recovery, there is growing recognition of the importance of considering other outcomes^71^. There are only a few studies of interventions designed to support recovery over the longer term (a notable exception is the Recovery Management Checkup)^74^. It is important to better understand how social identity evolves and changes over the course of recovery, with an emphasis on the emergence of strengths and pro-social connections as supportive recovery infrastructure^27,75,76^.

## Theme 3: Bridging the gap between implementation science and implementation practice

Methadone has been an effective treatment for OUD for more than 50 years, buprenorphine for nearly 20 years and extended release naltrexone for close to a decade. However, like many evidence-based interventions, these treatments are often not implemented in routine clinical settings. Likewise, a strong evidence-base for programs that prevent non-opioid drug use exists; however, evidence-based prevention interventions have not been widely implemented^3^^,4^. To make progress in addressing the opioid crisis, we must not only significantly shorten the lag for moving evidence-based prevention and treatment interventions from research into practice, but also understand the barriers to implementing them more broadly. This will require understanding the motives, goals and needs of payers, policy makers, organization and system decision makers, clinicians, drug companies and consumers, as all may play roles in determining which, whether and how interventions are delivered and sustained in practice^77^.

Within this theme, we highlight three areas of opportunity:

1. Considering scalability in implementation strategies.
2. User-centered design in implementation.
3. Modeling system complexity and looking beyond opioids.

### Considering scaling

A literature has emerged in the last 10 years seeking to identify effective approaches for implementing evidence-based interventions in routine clinical or community settings. Several promising approaches have emerged in prevention and treatment. For example, in prevention, Communities that Care (CTC) and PROSPER (PROmoting School-community-university Partnerships to Enhance Resilience) are effective approaches for engaging key stakeholders^78,79^ and implementing community-level prevention strategies. These approaches are being further adapted to be inclusive of the full continuum of prevention to treatment services. A limitation of some implementation models is that they often require very specific conditions to succeed, often with insufficient attention to how interventions can be scaled more broadly^80^. In particular, the role of intermediary organizations that provide technical support (e.g., SAMHSA’s Addiction Technology Transfer Centers) in replicating successful implementation models supporting scaling has not been sufficiently explored^81^.

While it is critical to provide rigorous scientific tests of implementation models, practical considerations must also be taken into account. These considerations include sustainability of financial and other resources^82,83^, and the long-term implications of potential findings. Research findings that are relevant and actionable should be made accessible and communicated to consumers and stakeholders. While publications in peer-reviewed journals and other forms of dissemination are important, the true measure of success is the adoption of interventions in routine clinical practice and community settings along with an improvement in outcomes among those who receive the interventions.

### User-centered design

Successfully designing scalable interventions and implementation supports will require engagement of a wide range of stakeholders— including patients, organization decision makers and leaders, payers, and policy makers. The concept of user-centered design in intervention development and implementation offers a useful perspective to overcome barriers to scalability. User-centered design approaches put the individuals who will benefit from a product or service at the center of its development, and these approaches begin intervention design with a focus on responsiveness to the needs of the end-user. This approach has shown promise for developing interventions that are highly responsive to individual needs and preferences.

Innovative designs offer powerful tools in thinking about how to optimize interventions around implementation and sustainability considerations^84^. User-centered design has not been sufficiently used in implementation science research, however. This lack of attention has led to disappointing results that should be avoided in future research on the design of both interventions and implementation models^63,64^. A recent study, for example, demonstrated that even an intervention that significantly improved patient outcomes and facilitated communication between patients and clinicians had very low uptake among clinicians and was not sustained past the end of the study. This was due in part to a lack of focus during development of the intervention on critical issues that determine sustainability from an organization perspective^33^. For long-term success in developing scalable, sustainable interventions, it is critical that intervention developers proactively consider the needs of both patients and providers from the outset of intervention design.

### Modeling system complexity and looking beyond opioids

Although randomized clinical trials are the gold standard in clinical research, they may be insufficient to address the current crisis given its changing nature, the diversity of populations affected, the inability to test all interventions in all populations, the complexity of the systems in which needs are identified and services are delivered, and the need to rapidly generate information that can be used by policy-makers and health system administrators^85^. Some well-intentioned policies may ultimately have unintended negative effects, and it is important to examine how to consider the multitudes of possible options^86^. For example, one recent study suggested that policies that target prevention of prescription opioid misuse might reduce opioid deaths by only 3–5%^87^, suggesting that targeting prescribing alone is not sufficient and that it is critical to balance resources across a wide variety of approaches. It is critical to develop and test models that can integrate data across a complex array of individual, organizational, and system level sources to provide valuable and efficient insights to guide policy and practice. For example, data on risk stratification could inform development of targeted interventions and strategies for tailoring them^2^.

Opioids are the focus of heightened concern now, but it is necessary to establish data collection and analysis infrastructures to anticipate and prevent future crises. Epidemiological data has revealed that in addition to the contribution of synthetic opioids to the rise in overdose fatalities, the past few years have seen a concomitant rise in fatalities associated with psychostimulant drugs (cocaine and methamphetamine) either by themselves or in combination with opioids^88^. Improvement in modeling capabilities should allow for a faster and better allocation of resources, and for improved planning of treatment and prevention services.

## Theme 4: Data to build cross-system collaborations and learning systems of care

Multiple agencies and systems in any given community interact with people who misuse opioids, individuals with OUD, and their families. This includes healthcare systems, specialty behavioral health services providers, education, justice, social service systems, and others. Often these agencies operate relatively independently. However, coordination and collaboration across these systems is essential to achieving successful, scalable solutions. Within this theme, we highlight barriers and opportunities in cross-system collaborations, and how these create an opportunity to build a learning system of care.

Within this theme, we highlight three areas of opportunity:

1. Building learning systems of care.
2. Developing common frameworks for understanding outcomes.
3. Thinking about data at a community level.

### Building learning systems of care

A learning health system has traditionally been defined as a health system “in which science, informatics, incentives, and culture are aligned for continuous improvement and innovation”^9^. Work on learning health systems has mostly focused on efforts internal to a single organization^89^. However, such a concept should also be advanced when multiple agencies and systems connect with the same individuals in a given community to address the variety of issues that emerge when all of these organizations interact and share resources and information. Advances in addressing the opioid crisis will require building learning systems of care. For the purposes of this discussion, we define a learning system of care as two or more organizations that must effectively interact and share data to serve the needs of a shared population of patients. The process of learning and improvement is one that is emergent across organizations and requires data sharing, collaboration, and common goals across these multiple, interacting organizations.

### Developing a common framework for understanding outcomes

**Fig. 1:**
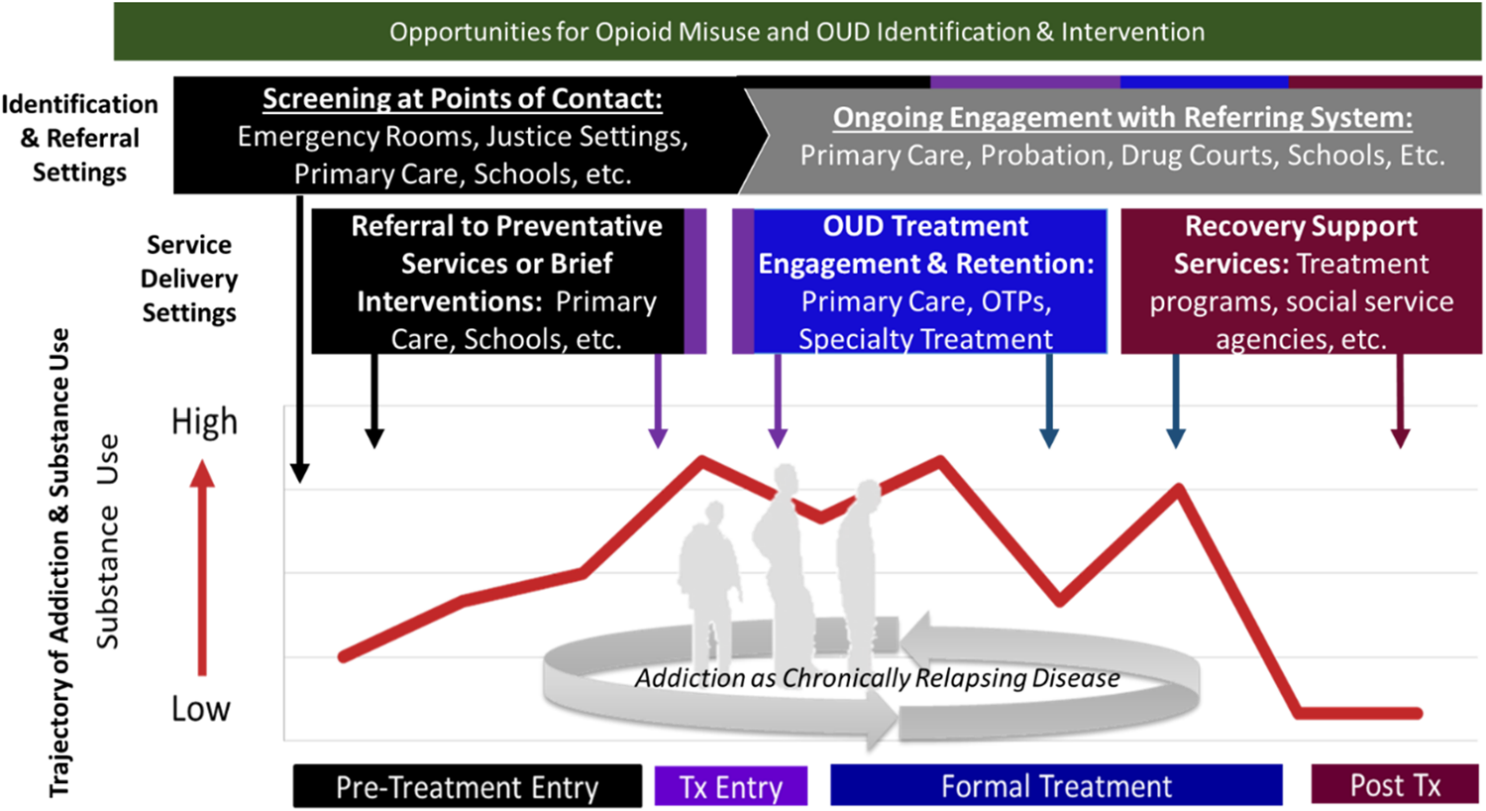
Opportunities for opioid misuse and OUD identification and intervention.

Achieving a vision of a learning system of care requires a shared set of outcomes of interest. The Opioid Use Disorder Cascade of Care^90–94^ has quickly become a common framework for thinking about key outcomes to be addressed in the opioid crisis. This framework is modeled after prior work in HIV^95–97^. Though the Opioid Use Disorder Cascade of Care specifically focuses on opioids (Fig. 1), it is straightforward to generalize this framework to other substances, outcomes and settings^98^. Implicit in many variations of a cascade of care framework is the importance of cross-system collaboration. That is, in many cases, the need for services (i.e., OUD treatment or prevention services) is often identified by a system or setting (e.g., emergency rooms, jails) that does not or cannot provide appropriate care (type, intensity, duration) for the identified need. When this is the case, cross-system collaboration is imperative to link individuals to the care needed and achieve the desired outcome of engaging an individual in evidence-based prevention or treatment services. Further, a single system can rarely address all needs of the individual. This framework is a useful starting point for thinking about how patients move across systems of care and is a powerful tool for planning comprehensive services and strategies, identifying gaps in service availability, and informing targets for interventions and services for a given system or setting. This perspective may be especially important as cost savings realized in one system (e.g., justice) may be transferred to another system (e.g., public behavioral health systems, health care system).

Cross-system collaborative efforts can be extraordinarily challenging. Challenges may include technical and regulatory barriers to information sharing^99^; differences in mission, goals and values^98^; communication challenges; lack of leadership buy-in; and, siloed funding. Despite these difficulties, recent work suggests that these barriers can be overcome and that the Cascade of Care framework can be used to facilitate positive, collaborative relationships across systems. For example, in NIDA’s JJ-TRIALS initiative^98^, juvenile justice systems and behavioral health providers in 36 communities partnered together and used a data-driven framework with the goal of increasing the proportion of youth with identified substance use needs receiving evidence-based services. The study examined the amount of facilitation necessary to successfully improve performance across the two systems and across the entire care cascade, a question with very pragmatic implications for how these kinds of efforts can be supported in the future. This cross-system collaboration is typical in identifying and treating substance use; Table 3 provides a general framework of key actors across the entire Cascade of Care.

**Table 3:** The OUD services cascade: key actors and challenges in The services cascade.

Cascade step	Step outcome	Key actors	Challenges
Screening	Need Identification	Providers in settings where people with opioid misuse and OUD present, such as Primary Care, Emergency Departments, Justice Settings, Mental Health Specialty, and School-based health centers.	• Referral is required for screening to take place • In some settings, screening for OUD may not considered a priority • Screening often must take place rapidly or in settings with competing demands
Need Identification and referral to services	*Need identified*	*Services referred to*	• Non-diagnostic instruments are often used • Referral and tracking across systems can be difficult • Patients may have complex treatment needs, including comorbid psychiatric conditions • Few evidence-based approaches to facilitate successful transition across settings • Referrals may be both intra-organizational or inter-organizational • Referral may not always be possible due to lack of relationships or other barriers • Availability of services may be unknown
	None	N/A	
	Preventative or low intensity services indicated	Brief interventions prevention services settings	
	Behavioral health services needed	Outpatient specialty treatment settings	
	Pharmacotherapy needed	OUD treatment settings (see Table 2)	
	Intensive services needed	Inpatient treatment settings	
	Other services	E.g., Mental health, HIV, housing	
Initiation of and retention in services	*Type of service*	*Key actors*	• Brief interventions for OUD may not be effective • Can be difficult to train clinicians or peers to consistently deliver brief interventions
	Brief interventions	Clinicians or peers properly trained in brief interventions	
	Prevention services	E.g., educational settings, occupation/workplace, faith-based organizations, social services.	• Preventative services do not exist in a well-established service ecosystem • Few targeted prevention interventions exist specifically for opioids
	Outpatient addiction treatment	See Table 2 for care settings for medications. Care may occur in a single integrated care setting, or more commonly, multiple discrete care settings (e.g., psychiatric treatment, treatment of other SUD)	• Diagnostic assessment must often be completed to determine appropriate referrals • Integrated care is desirable to address comorbid conditions including psychiatric services, but this goal is not always easily achieved • Mortality risk is elevated after inpatient care, and care is not always well coordinated • Bi-directional information sharing can be challenging
	OUD treatment settings		
	Inpatient addiction treatment settings		
	Other services (e.g., HIV)		

### Data at the community level

Connecting data across multiple systems of care to understand how individuals may move across systems can drive new insights on how to optimally allocate resources and establish successful cross-system collaborations. A recent analysis in Maryland found that most individuals who experienced a fatal overdose had been captured in opioid prescription databases, hospital records, or criminal justice databases^100^.

Data collection, surveillance, and analysis systems that allow for real-time reporting and open access sharing of information across systems are critical to understanding the impacts of service models and collaborative efforts and must incorporate a feedback loop that accounts for and characterizes the various trajectories and systems within which they operate. Additional research that demonstrates how new insights can be generated by connecting multiple data sources at the state and local level is needed. A long-term goal is to identify viable models for standardizing data nationally in ways that can generate broader insights. There is need for a national approach that can, on an ongoing basis, coordinate comprehensive data from multiple systems and sources, and generate predictive models of heterogeneous subpopulations to evaluate the impacts of interventions (e.g., policy, behavioral, pharmaceutical, enforcement) and collaborative efforts across multiple systems. This would provide invaluable data and tools necessary to identify what is or is not working for whom^101,102^.

Models of integrated data systems can be utilized to drive community-level prevention and treatment interventions. CTC and PROSPER are examples of data-driven, community and cross-system collaborative models that include a learning system of care approach^78,79^ and that can be informative in developing a standardized national program.

## Conclusion

OUD is a complex disease that exists in an equally complex ecosystem. In order to develop appropriate prevention and treatment interventions to address the opioid crisis, it is important to better understand how risk profiles and opioid misuse and OUD pathways differ across individuals and populations. A translational approach with ongoing dialogue across epidemiology, prevention, treatment and other disciplines is needed to understand these diverse trajectories, and to ensure practice and policy are drawing from the most comprehensive evidence base. A primary goal of services and implementation research on opioid misuse and OUD is to generate evidence of how to optimize the delivery of effective and sustainable prevention and treatment interventions and strategies to individuals, families, and communities. The four themes outlined above provide us with a roadmap on how to achieve these goals.

## References

[1] Blanco C, Volkow ND (2019). Management of opioid use disorder in the USA: present status and future directions. Lancet.

[2] Blanco C, Wall M, Liu S, Olfson M (2019). Towards a comprehensive developmental model of prescription opioid use disorder. J. Clin. Psychiatry.

[3] National Research Council and Institute of Medicine. *Preventing Mental, Emotional, and Behavioral Disorders Among Young People: Progress and Possibilities*. (The National Academies Press*,* 2009). 20662125

[4] U.S. Department of Health and Human Services (HHS) Office of the Surgeon General. *Facing Addiction in America: The Surgeon General’s Report on Alcohol, Drugs, and Health* (HHS, 2016). 28252892

[5] Spoth R (2013). Longitudinal effects of universal preventiLongitudinal effects of universal preventive intervention on prescription drug misuse: three RCTs with late adolescents and young adults. Am. J. Public Health.

[6] Blanco C (2016). Pain as a predictor of opioid use disorder in a nationally representative sample. Am. J. Psychiatry.

[7] Han B (2017). Prescription opioid use, misuse, and use disorders in U.S. adults: 2015 National Survey on Drug Use and Health. Ann. Intern. Med..

[8] Han B, Compton WM, Blanco C, Jones CM (2018). Correlates of prescription opioid use, misuse, use disorders, and motivations for misuse among US adults. J. Clin. Psychiatry.

[9] Institute of Medicine. *The Learning Healthcare System: Workshop Summary* (The National Academies Press*,* 2007). 21452449

[10] Quinones, S. *Dreamland: The True Tale of America’s Opiate Epidemic* (Bloomsbury Publishing, USA, 2015).

[11] Hari, J. *Chasing the Scream: The First and Last Days of the War on Drugs* (Bloomsbury Publishing, USA, 2015).

[12] Case A, Deaton A (2015). Rising morbidity and mortality in midlife among white non-Hispanic Americans in the 21st century. Proc. Natl Acad. Sci. USA.

[13] Martins SS (2017). Changes in US lifetime heroin use and heroin use disorder: prevalence from the 2001-2002 to 2012-2013 national epidemiologic survey on alcohol and related conditions. JAMA Psychiatry.

[14] Green TC (2018). Postincarceration fatal overdoses after implementing medications for addiction treatment in a statewide correctional system. JAMA Psychiatry.

[15] Rich JD (2015). Methadone continuation versus forced withdrawal on incarceration in a combined US prison and jail: a randomised, open-label trial. Lancet.

[16] Dowell D, Haegerich TM, Chou R (2016). CDC Guideline for prescribing opioids for chronic pain—United States, 2016. JAMA.

[17] Dowell D, Haegerich T, Chou R (2019). No shortcuts to safer opioid prescribing. New England. J. Med..

[18] Von Korff M (2016). United States national pain strategy for population research: concepts, definitions, and pilot data. J. Pain..

[19] Patel MS, Volpp KG, Asch DA (2018). Nudge units to improve the delivery of health care. N. Engl. J. Med..

[20] Fox AD, Chamberlain A, Sohler NL, Frost T, Cunningham CO (2015). Illicit buprenorphine use, interest in and access to buprenorphine treatment among syringe exchange participants. J. Subst. Abus. Treat..

[21] Williams AR, Bisaga A (2016). From AIDS to opioids-how to combat an epidemic. N. Engl. J. Med..

[22] Ghosh D (2017). Social network strategies to address HIV prevention and treatment continuum of care among at-risk and HIV-infected substance users: a systematic scoping review. AIDS Behav..

[23] Latkin CA, Knowlton AR (2015). Social network assessments and interventions for health behavior change: a critical review. Behav. Med..

[24] Moos RH (2005). Iatrogenic effects of psychosocial interventions for substance use disorders: prevalence, predictors, prevention. Addiction.

[25] Kuntsche SKE (2016). Parent-based interventions for preventing or reducing adolescent substance use-a systematic literature review. Clin. Psychol. Rev..

[26] Hogue A, Liddle HA (2009). Family‐based treatment for adolescent substance abuse: controlled trials and new horizons in services research. J. Fam. Ther..

[27] Best D (2016). Overcoming alcohol and other drug addiction as a process of social identity transition: the social identity model of recovery (SIMOR). Addiction Res. Theory.

[28] Harocopos A, Goldsamt LA, Kobrak P, Jost JJ, Clatts MC (2009). New injectors and the social context of injection initiation. Int. J. Drug Policy.

[29] Braciszewski, J. M. et al. Developing a tailored substance use intervention for youth exiting foster care. *Child Abuse Neglect* 211–221 (2018). 10.1016/j.chiabu.2018.01.013 PMC5857233 29367098

[30] Behrendt S, Buhringer G, Hofler M, Lieb R, Beesdo-Baum K (2017). Prediction of incidence and stability of alcohol use disorders by latent internalizing psychopathology risk profiles in adolescence and young adulthood. Drug Alcohol Depend..

[31] Kennedy B (2019). Low stress resilience in late adolescence and risk of smoking, high alcohol consumption and drug use later in life. J. Epidemiol. Community Health.

[32] Compton WM, Jones CM, Baldwin GT, Harding F, Wargo EM (2019). Using prevention science principles to address the U.S. opioid crisis. Am. J. Public Health.

[33] Quanbeck A (2018). Implementing a mobile health system to integrate the treatment of addiction into primary care: a hybrid implementation-effectiveness study. J. Med. Internet Res..

[34] Ashford RD, Lynch K, Curtis B (2018). Technology and social media use among patients enrolled in outpatient addiction treatment programs: cross-sectional survey study. J. Med. Internet Res..

[35] D’Agostino AR (2017). Social networking online to recover from opioid use disorder: a study of community interactions. Drug Alcohol Depend..

[36] Cavazos-Rehg PA (2020). Delivering information about medication assisted treatment to individuals who misuse opioids through a mobile app: a pilot study. J. Public Health.

[37] Institute of Medicine (US) Committee on Quality of Health Care in America. Crossing the Quality Chasm: A New Health System for the 21st Century. Washington (DC): National Academies Press (US); 2001. 25057539

[38] Liang H (2017). The patient-centered care and receipt of preventive services among older adults with chronic diseases: a nationwide cross-sectional study. Inq. J. Med. Care Organ. Provis. Financ..

[39] Marchand K (2018). Patient-centred care for addiction treatment: a scoping review protocol. BMJ Open.

[40] Pincus, H. A. & Blanco, C. The opioid crisis in America: an overview. (eds. Weil, A. R. & Dolan, R.) Confronting our nation’s opioid crisis, a report of the Aspen Health Strategy Group. 23–46 (Aspen Institute, 2017).

[41] Ford JA, Pomykacz C, Veliz P, McCabe SE, Boyd CJ (2018). Sports involvement, injury history, and non-medical use of prescription opioids among college students: an analysis with a national sample. Am. J. Addict..

[42] Fleary SA, Heffer RW, McKyer ELJ (2013). Understanding nonprescription and prescription drug misuse in late adolescence/young adulthood. J. Addict..

[43] Schepis TS, McCabe SE (2016). Trends in older adult nonmedical prescription drug use prevalence: results from the 2002-2003 and 2012-2013 National Survey on Drug Use and Health. Addict. Behav..

[44] Davis MA, Lin LA, Liu H, Sites BD (2017). Prescription opioid use among adults with mental health disorders in the United States. J. Am. Board Fam. Med..

[45] Wall MC-PK, Hu MC, Feng T, Griesler P, Kandel DB (2018). Nonmedical prescription opioids and pathways of drug involvement in the US: generational differences. Drug Alcohol Depend.

[46] Curtis BL, Ashford RD, Magnuson KI, Ryan-Pettes SR (2019). Comparison of smartphone ownership, social media use, and willingness to use digital interventions between generation Z and millennials in the treatment of substance use: cross-sectional questionnaire study. J. Med. Internet Res,.

[47] Carroll JJ, Marshall BDL, Rich JD, Green TC (2017). Exposure to fentanyl-contaminated heroin and overdose risk among illicit opioid users in Rhode Island: a mixed methods study. Int. J. Drug Policy.

[48] Macmadu A, Carroll JJ, Hadland SE, Green TC, Marshall BD (2017). Prevalence and correlates of fentanyl-contaminated heroin exposure among young adults who use prescription opioids non-medically. Addict. Behav..

[49] Brothers TD, Bonn M (2019). Patient-centred care in opioid agonist treatment could improve outcomes. Can. Med. Assoc. J..

[50] Carlson RG, Nahhas R, Martins SS, Daniulaityte R (2015). Predictors of transition to heroin use among initially non-opioid dependent illicit pharmaceutical opioid users: a natural history study. Drug Alcohol Depend.

[51] Teruya C (2014). Patient perspectives on buprenorphine/naloxone: a qualitative study of retention during the starting treatment with agonist replacement therapies (START) study. J. Psychoact. Drugs.

[52] Webster LR (2017). Risk factors for opioid-use disorder and overdose. Anesthesia Analgesia.

[53] McKnight, C. D. J. D. Being “hooked up” during a sharp increase in the availability of illicitly manufactured fentanyl: adaptations of drug using practices among people who use drugs (PWUD) in New York City. *Int. J. Drug Policy* **60**, 82–88 (2018). 10.1016/j.drugpo.2018.08.004 PMC6457118 30176422

[54] Peiper, N. C. C. S., Vincent, L. B., Ciccarone, D., Kral, A. H. & Zibbell, J. E. Fentanyl test strips as an opioid overdose prevention strategy: findings from a syringe services program in the Southeastern United States. *Int. J. Drug Policy* **63**, 122–128 (2019). 10.1016/j.drugpo.2018.08.007 30292493

[55] Coviello DM (2013). Does mandating offenders to treatment improve completion rates?. J. Subst. Abus. Treat..

[56] Barry MJ, Edgman-Levitan S (2012). Shared decision making—the pinnacle of patient-centered care. N. Engl. J. Med..

[57] Shay LA, Lafata JE (2015). Where is the evidence? A systematic review of shared decision making and patient outcomes. Med. Decis. Mak..

[58] Mojtabai R, Mauro C, Wall MM, Barry CL, Olfson M (2019). Medication treatment for opioid use disorders in substance use treatment facilities. Health Aff..

[59] amfAR's Opioid & Health Indicators database. *National Opioid epidemic: facilities providing substance abuse services*. http://opioid.amfar.org/indicator/SA_fac2018. Accessed 2 Jan 2020 (2018).

[60] Bruneau J (2018). Management of opioid use disorders: a national clinical practice guideline. Can. Med. Assoc. J..

[61] Saucier, R., Wolfe, D. & Dasgupta, N. Review of case narratives from fatal overdoses associated with injectable naltrexone for opioid dependence. *Drug Safety* **41**, 981–988 (2018). 10.1007/s40264-018-0653-3 29560596

[62] Lee JD (2018). Comparative effectiveness of extended-release naltrexone versus buprenorphine-naloxone for opioid relapse prevention (X: BOT): a multicentre, open-label, randomised controlled trial. Lancet.

[63] Hell ME, Miller WR, Nielsen B, Nielsen AS (2018). Is treatment outcome improved if patients match themselves to treatment options? Study protocol for a randomized controlled trial. Trials.

[64] Lei H, Nahum-Shani I, Lynch K, Oslin D, Murphy SA (2012). A “SMART” design for building individualized treatment sequences. Annu. Rev. Clin. Psychol..

[65] Almirall D, Compton SN, Gunlicks-Stoessel M, Duan N, Murphy SA (2012). Designing a pilot sequential multiple assignment randomized trial for developing an adaptive treatment strategy. Stat. Med..

[66] King, C. A. et al. Association of the youth-nominated support team intervention for suicidal adolescents with 11-to 14-year mortality outcomes: secondary analysis of a randomized clinical trial. *JAMA Psychiatry* **76**, 492–498 (2019). 10.1001/jamapsychiatry.2018.4358 PMC6495350 30725077

[67] Ayers JW, Ribisl KM, Brownstein JS (2011). Tracking the rise in popularity of electronic nicotine delivery systems (electronic cigarettes) using search query surveillance. Am. J. Preventive Med..

[68] Young, S. D., & Heinzerling, K. The harnessing online peer education (HOPE) intervention for reducing prescription drug abuse: a qualitative study. *J. Substance Use* **22**, 592–596 (2017). 10.1080/14659891.2016.1271039 PMC5854394 29551953

[69] Mark, T. L., O’Brien, J., Mendell, G. & McLellan, A. T., Arsenault S. Improving Addiction Treatment With Consumer Report Cards. Health Affairs Blog. https://www.healthaffairs.org/do/10.1377/hblog20180102.514756/full/ (2018).

[70] Development services group I. Office of juvenile justice and delinquency prevention-model programs guide. *Positive Youth Development” Literature Review* (2014).

[71] Blanco C, Okuda M, Wang S, Liu SM, Olfson M (2014). Testing the drug substitution switching-addictions hypothesis. A prospective study in a nationally representative sample. JAMA Psychiatry.

[72] Flórez-Salamanca L (2013). Probability and predictors of cannabis use disorders relapse: results of the national epidemiologic survey on alcohol and related conditions (NESARC). Drug Alcohol Depend..

[73] Garcia-Rodriguez O (2013). Probability and predictors of relapse to smoking: results of the national epidemiologic survey on alcohol and related conditions (NESARC). Drug Alcohol Depend..

[74] Dennis ML, Scott CK, Funk R, Foss MA (2005). The duration and correlates of addiction and treatment careers. J. Subst. Abus. Treat..

[75] Dingle GA, Stark C, Cruwys T, Best D (2015). Breaking good: breaking ties with social groups may be good for recovery from substance misuse. Br. J. Soc. Psychol..

[76] Dingle G (2014). Social and transitional identity: exploring social networks and their significance in a therapeutic community setting. Therapeutic Communities.

[77] Hadland SE, Cerda M, Li Y, Krieger MS, Marshall BDL (2018). Association of pharmaceutical industry marketing of opioid products to physicians with subsequent opioid prescribing. JAMA Intern. Med..

[78] Crowley, D. M., Greenberg, M. T., Feinberg, M. E., Spoth, R. L., & Redmond, C. R. The effect of the PROSPER partnership model on cultivating local stakeholder knowledge of evidence-based programs: a five-year longitudinal study of 28 communities. *Prevent. Sci.* **13**, 96–105 (2012). 10.1007/s11121-011-0250-5 PMC3350746 21986990

[79] Arthur, M. W. et al. Implementation of the communities that care prevention system by coalitions in the community youth development study. *J. Community Psychology* **38**, 245–258 (2010). 10.1002/jcop.20362 PMC3244354 22199409

[80] Aarons GA, Sklar M, Mustanski B, Benbow N, Brown CH (2017). “Scaling-out” evidence-based interventions to new populations or new health care delivery systems. Implement. Sci..

[81] Proctor E (2019). Intermediary/purveyor organizations for evidence-based interventions in the US child mental health: characteristics and implementation strategies. Implement. Sci..

[82] Greenberg, M. T. et al. Factors that predict financial sustainability of community coalitions: five years of findings from the PROSPER partnership project. *Prevent. Sci.* **16**, 158–167 (2015). 10.1007/s11121-014-0483-1 PMC4186916 24706195

[83] Jonkman, H. B., et al. Communities that care, core elements and context: research of implementation in two countries. *Social Dev. Issues* 42–57 (2009). PMC2712284 19617929

[84] Collins, L. M., Kugler, K. C., & Gwadz, M. V. Optimization of multicomponent behavioral and biobehavioral interventions for the prevention and treatment of HIV/AIDS. *AIDS Behav.* S197–S214 (2016). 10.1007/s10461-015-1145-4 PMC4715714 26238037

[85] Blanco C, Compton WM, Weiss SR (2017). From epidemiology to interventions and back. Am. J. Psychiatry.

[86] Pitt AL, Humphreys K, Brandeau ML (2018). Modeling health benefits and harms of public policy responses to the US opioid epidemic. Am. J. Public Health.

[87] Chen Q (2019). Prevention of prescription opioid misuse and projected overdose deaths in the United States. JAMA Netw. Open.

[88] Centers for Disease Control and Prevention. 2018 annual surveillance report of drug-related risks and outcomes—United States. surveillance special report 2. *Centers for Disease Control and Prevention*, *US Department of Health and Human Services* (2018).

[89] Abraham, E. et al. Generating Knowledge from Best Care: advancing the continuously learning health system. NAM perspectives. *Discussion Paper* (National Academy of Medicine, Washington: DC, 2016).

[90] Socias ME, Volkow N, Wood E (2016). Adopting the ‘cascade of care’ framework: an opportunity to close the implementation gap in addiction care?. Addiction.

[91] Williams AR (2018). Developing an opioid use disorder treatment cascade: a review of quality measures. J. Subst. Abus. Treat..

[92] Williams AR, Nunes EV, Bisaga A, Levin FR, Olfson M (2019). Development of a cascade of care for responding to the opioid epidemic. Am. J. Drug Alcohol Abus..

[93] Dennis ML (2019). Operationalizing a behavioral health services cascade of care model: lessons learned from a 33-site implementation in juvenile justice community supervision. Federal Probation..

[94] Williams AR, Nunes EV, Bisaga A, Levin FR, Olfson M (2019). Development of a Cascade of Care for responding to the opioid epidemic. Am. J. Drug Alcohol Abus..

[95] Gardner EM, McLees MP, Steiner JF, Del Rio C, Burman WJ (2011). The spectrum of engagement in HIV care and its relevance to test-and-treat strategies for prevention of HIV infection. Clin. Infect. Dis..

[96] Greenberg AE (2009). Fighting HIV/AIDS in Washington, D.C. Health Aff..

[97] MacCarthy S (2015). The HIV care cascade: models, measures and moving forward. J. Int. AIDS Soc..

[98] Belenko S (2017). The juvenile justice behavioral health services cascade: a new framework for measuring unmet substance use treatment services needs among adolescent offenders. J. Subst. Abus. Treat..

[99] McCarty D, Rieckmann T, Baker RL, McConnell KJ (2017). The perceived impact of 42 CFR part 2 on coordination and integration of care: a qualitative analysis. Psychiatr. Serv..

[100] Eisenberg MD (2019). Use of opioid overdose deaths reported in one stateas criminal justice, hospital, and prescription databases to identify risk of opioid fatalities. JAMA Int. Med.

[101] Bernard CL, Owens DK, Goldhaber-Fiebert JD, Brandeau ML (2017). Estimation of the cost-effectiveness of HIV prevention portfolios for people who inject drugs in the United States: a model-based analysis. PLoS Med..

[102] David C (2017). A human judgment approach to epidemiological forecasting. PLoS Comput. Biol..

[103] Trujols J (2017). Patient perception of methadone dose adequacy in methadone maintenance treatment: The role of perceived participation in dosage decision. Patient Educ. Counseling.

